# Inside the Black Box: Examining the Interconnectivity of Components of Integrated Person-Centred Care for Vulnerable Groups Through a Realist Lens

**DOI:** 10.5334/ijic.8655

**Published:** 2024-08-08

**Authors:** Anam Ahmed

**Affiliations:** 1 Panaxea, Den Bosch, The Netherlands

**Keywords:** integrated care, person-centred care, vulnerable groups, realist research

## Abstract

Integrated person-centred care (IPCC) for vulnerable groups is complex and multifaceted and cannot be reduced to simple cause-and-effect relationships. The effectiveness varies across settings due to differing contexts and mechanisms. By applying realist research, this dissertation examines the relationships between the context in which IPCC for vulnerable groups in the Netherlands is applied, the mechanisms by which IPCC (does not) work(s), and the outcomes resulting from this interaction. The findings provide deeper insights into interrelatedness of items that influence effectiveness of IPCC, emphasizing the significance of understanding their interplay and recognizing that they form a larger interconnected system rather than acting independently.

## Introduction

Current healthcare systems, still largely focused on single diseases and acute problems, are confronted with increasing pressure, personnel shortages, and rising costs [1,2,3]. As a result they are inadequate for delivering comprehensive care to the entire population [4,5,6,7,8,9]. Vulnerable groups, such as older individuals, those with low health literacy, and diverse ethnic backgrounds, face greater health risks and limited access to services [10,11,12]. Integrated person-centred care (IPCC) is seen as the optimal care delivery model for improving health outcomes [13,14,15]. It emphasizes holistic client needs and involves a network of health (and social) care providers and organizations [16,17]. Primary care is acknowledged the most efficient and cost-effective setting for IPCC [13]. However, challenges like care coordination, insufficient training of care providers, incompatible information and communication technology systems, and funding issues hinder IPCC [18]. Moreover, scientific literature on the effectiveness of IPCC is inconclusive, partly due to the heterogeneity in outcomes [19,20,21,22,23,24,25,26].

### Objective

The overarching objective of the dissertation is to examine the relationships between the context in which IPCC for vulnerable groups in the Netherlands is applied, the mechanisms by which the complex programme (does not) work, and the outcomes resulting from this interaction.

### Methodology

To provide a more detailed understanding of the interrelatedness of relevant items that influence the effectiveness of IPCC, the ‘realist research’ approach is used in this dissertation. Realist research aims to develop a programme theory that explains how, why, and for whom a complex programme is expected to work in what circumstances [27,28,29,30,31,32]. The initial programme theory, middle-range programme theory, and refined programme theory represent different stages of developing and refining the understanding of how a program works.

The dissertation consists of two studies initiated by the National Health Care Institute in the Netherlands. The first study focused on integrated care programmes (ICPs) for community-dwelling frail older people, where a rapid realist review (RRR) was conducted to identify context items (C), mechanisms (M), and outcomes (O) in the international literature (Chapter 2) and was tested and refined through a Delphi study with HCPs (Chapter 3).

In the second study, person-centred care was examined for socially vulnerable groups such as individuals with limited health literacy skills and those from diverse ethnic and socioeconomic backgrounds. A realist review was conducted to develop a middle-range programme theory (Chapter 4), followed by focus group discussions (FGDs) to refine this theory for the ‘Dutch setting’ and assess the consensus on the relevance of items derived from the RRR (Chapter 5).

## Findings

### Key items across programme theories

Several key items are mentioned by both programme theories regarding IPCC for vulnerable groups. The benefit of examining multiple program theories in this dissertation is that it can provide a more comprehensive and nuanced understanding of how IPCC works for vulnerable groups and in what circumstances. Both program theories underscore the significance of tailoring care to address the unique needs and preferences of individuals (O). HCPs should strive to deliver holistic care (M) that encompasses the physical, mental, and social aspects of health, recognizing the diversity among individuals (C). The need for comprehensive training of HCPs (C) is emphasized in both programme theories. Equipping professionals with effective communication skills (M) and interprofessional collaboration competencies (M) enables them to deliver high-quality care (O). The recognition of collaboration within multidisciplinary teams and across different domains (C) also emerges as a common theme. The program theories highlight the essential role of teamwork in achieving optimal care coordination (M), resulting in improved satisfaction of individuals (O). By fostering a clear division in roles and responsibilities (C), as well as promoting awareness of each other’s expertise (C), HCPs can effectively collaborate (M) to address complex and diverse care needs. Effective tailored communication (M) serves as a cornerstone in both program theories. By involving individuals in their care process (M) and empowering individuals (M) shared decision-making (M) can be facilitated. Both program theories acknowledge the transformative potential of ICT-systems (C). By integrating technology into healthcare practices, accessibility of care (C, O) is enhanced, facilitating seamless communication among HCPs, individuals, and caregivers. Through information sharing (C), health-related social networks (C), and electronic access to guidelines and protocols (C), individuals are empowered to actively engage in their care journey (O). The program theories underscore the role of education to people (C) and active involvement in their care process (M). By providing self-management support to people (M) and encouraging them to ask questions (C) their self-efficacy can be enhanced (O). Shared goal setting (C), personalized care planning (C), and a shared vision (C) can improve their overall health and well-being (O).

## Implications for integrated care and future research

### Research

The dissertation highlights the need for additional realist research to explore the theoretical underpinning among context items, mechanisms, and outcomes (C, M, O) for developing robust program theories. It provides valuable insights by identifying an optimal combination of these elements for IPCC, acknowledging its variability across settings. More data on the health and healthcare use by underrepresented vulnerable groups is needed, along with stakeholder involvement for setting-specific validation of items. Further investigation is required for items where no consensus was reached to understand their deemed irrelevance and potential conceptual differences in IPCC in the Netherlands compared to other settings. Lastly, it is recommended to assess the collective implementation and outcomes of the items in the Dutch setting to validate the program theories.

### Practice

At the micro level, HCPs should prioritize training in empathic and culturally sensitive care, effective communication, and holistic approaches. Encouraging interprofessional collaboration early in training, building relationships with primary, secondary, and social care partners, and proactive engagement from individuals and communities can enhance care experiences.

Organizations at the meso level should have shared ambitions and collaborate in policy, commissioning, and implementing IPCC. Collaboration with relevant partners can enhance connections, such as through bundled budgets and prevention agreements.

Involving individuals in care design, tailored approaches for vulnerable groups, and long-term financing to reduce health inequalities are essential.

In the Netherlands, regional collaboration can be promoted through initiatives such as “regional overviews and approaches” (regiobeelden en regioplannen) as part of the “Right Care at the Right Place” movement.

On a macro level, stakeholders directing policy, financing, and care organization must prioritize promoting and implementing IPCC with attention to diversity in policy. This includes developing multidisciplinary care standards, quality indicators for person-centredness, and removing financial barriers for integrated cross-domain care. Initiatives like the Appropriate Care programme and the Integral Care Agreement in the Netherlands underscore the importance of high-quality, accessible, and affordable care for diverse patient populations.

## Conclusion

IPCC for vulnerable groups is complex and multifaceted and cannot be reduced to simple cause-and-effect relationships. The effectiveness varies across settings due to differing contexts and mechanisms. The findings of this dissertation provide deeper insights into interrelatedness of items that influence effectiveness of IPCC, emphasizing the significance of understanding their interplay and recognizing that they form a larger interconnected system rather than acting independently.

**ISBN:** 978-94-6483-550-2.
